# Video recording emergency care and video-reflection to improve patient care; a narrative review and case-study of a neonatal intensive care unit

**DOI:** 10.3389/fped.2022.931055

**Published:** 2022-08-04

**Authors:** Veerle Heesters, Ruben Witlox, Henriette A. van Zanten, Sophie J. Jansen, Remco Visser, Veerle Heijstek, Arjan B. Te Pas

**Affiliations:** Division of Neonatology, Department of Paediatrics, Willem-Alexander Children’s Hospital, Leiden University Medical Center, Leiden, Netherlands

**Keywords:** video recording, emergency care, video review, clinical audit, quality improvement, video-reflection, videotape recording

## Abstract

As the complexity of emergency care increases, current research methods to improve care are often unable to capture all aspects of everyday clinical practice. Video recordings can visualize clinical care in an objective way. They can be used as a tool to assess care and to reflect on care with the caregivers themselves. Although the use of video recordings to reflect on medical interventions (video-reflection) has increased over the years, it is still not used on a regular basis. However, video-reflection proved to be of educational value and can improve teams’ management and performance. It has a positive effect on guideline adherence, documentation, clinical care and teamwork. Recordings can also be used for video-reflexivity. Here, caregivers review recordings together to reflect on their everyday practice from new perspectives with regard to context and conduct in general. Although video-reflection in emergency care has proven to be valuable, certain preconditions have to be met and obstacles need to be overcome. These include gaining trust of the caregivers, having a proper consent-procedure, maintaining confidentiality and adequate use of technical equipment. To implement the lessons learned from video-reflection in a sustainable way and to continuously improve care, it should be integrated in regular simulation training or education. This narrative review will describe the development of video recording in emergency care and how video-reflection can improve patient care and safety in new ways. On our own department, the NICU at the LUMC, video-reflection has already been implemented and we want to further expand this. We will describe the use of video-reflection in our own unit. Based on the results of this narrative review we will propose options for future research to increase the value of video-reflection.

## Introduction

Video recordings offer the possibility to visualize clinical care in a comprehensive and objective way. Due to the emergent nature of critical care, recordings proved to be a valuable addition in evaluation and improvement (1, 2). The environment in emergency care can be chaotic and protocols are not always followed. This makes it difficult to make the necessary quality improvements. Recordings can be used to identify new areas of improvement in emergency care (3). Reflection by caregivers show them the aspects of care they enact without conscious awareness. The clinicians then have the ability to reflect on their own habits and intervene to improve future clinical outcomes (4). Video-reflection is affordable and relatively easy to initiate with technical equipment available worldwide. So far, reflection on recordings of emergency care has been used for studies in neonatology, trauma care, obstetrics, pediatrics and in cardiac arrest teams, but not routinely during daily care (5). Although these studies show video-reflection has the potential to improve teamwork and quality of patient care, it remains unclear what different types of video-reflection are often used and how they differ in improving quality of care. The aim of this narrative review is to describe the different applications of video recording in clinical care and their effect. We discuss preconditions and obstacles of video-reflection and explore how video-reflection can be used more routinely to improve care. On our own department, the NICU at the Leiden University Medical Center, we have already implemented video-reflection of neonatal resuscitations. At the end of this review we will describe the use of video-reflection in our own unit. In light of the results of this narrative review, we will propose how future studies can enhance video-reflection on our neonatal department.

## Materials and methods

This narrative review is based on a literature search which was performed in collaboration with a medical information specialist. To identify all relevant publications about video-reflection or audits of interventions in healthcare we performed searches in the bibliographic databases PubMed, EMBASE.com and Emcare (*via* Ovid) from inception to October 7th, 2021. Search terms included controlled terms (MeSH in PubMed and Emtree in Embase/Emcare) as well as free text terms. Search terms expressing ‘video’ were used in combination with search terms comprising ‘clinical audit.’ The primary researcher (VH) conducted a screening and made a list of articles that would be full-text assessed. This included all articles on different methods of video-reflection in emergency care. The literature was supplemented with studies found by reviewing the references of the included studies. A search strategy can be found in the Supplementary material.

## Using recordings for assessing quality of care

### Compliance to guidelines

Video recordings can help compare work-as-done with work-as-imagined. Work-as-done is a term describing the daily activities of everyday clinical work. Healthcare workers adapt and adjust their actions according to the specific situations they face and the needs of the patients they are caring for. Work-as-imagined describes the manner in which it is assumed that people will do their work. It refers to the way people who make guidelines or regulations believe the work should be carried out. There may be a considerable difference between what people are expected to do and what they actually do (6). Recordings can be used to assess the actual guideline adherence. Studies using video recordings in emergency care have shown that caregivers often deviate from the guidelines (7–10). These insights give the opportunity to evaluate and discuss the prevailing guidelines and adapt them where needed (8, 11). Although more studies are needed to measure the effect of reevaluating guidelines, assessment of recordings of clinical care can emphasize the continuous need for reinforcement of adherence (7, 10, 12).

As a result of this, protocols can also be expanded or adjusted. Taylor et al. used video recordings to create specific time goals for key steps in trauma resuscitation to reduce the total amount of time spent in the trauma bay. Plan-Do-Study-Act cycles were used to define time goals in order to improve quality (13). Aydon et al. (14) assessed leadership and handover during the first hour after admission to the NICU, which resulted in implementation of extra guidelines for handover. Furthermore, video recordings have been used to evaluate the rate of compliance to the use of protective clothing and accessories to prevent exposure to blood or body fluids (2). Even differences between centers in various countries, with their own local guidelines, can be made visible by assessing video recordings (15).

### Non-technical skills

Guidelines focus mainly on technical aspects of care. However, it is also important to include non-technical aspects for a good quality of care. Studies show non-technical aspects, like communication, leadership and teamwork, can be assessed well with video recordings (12, 16–19). Williams et al. (20) used the Crew Resource Model from the aviation industry to study the relationship between teamwork behaviors and errors using video recordings. They observed that more vigilant teams committed fewer errors. Nadler et al. (21) used video recordings of neonatal resuscitations and scored them on teamwork, guideline adherence and temporal control of the resuscitation procedure.

## Using recordings for video-reflection

Video recordings can also be used to allow caregivers to reflect on their practice and evaluate the care given. This so called video-reflection enables caregivers to see what actually happened during a procedure and compares this with their recollection of what happened. Videotaping care has the benefit that it allows for assessment at any hour, without intrusion of an observer (22). The camera is an impartial observer and can create a more detailed recollection of interventions than the caregivers themselves (17). This is confirmed by Schilleman et al. in a study showing that documentation of neonatal resuscitation in the medical file is often inaccurate and differs from what is seen in video recordings of the same resuscitation. Recordings can clarify documentation because they give an unbiased view (23). Next to that, recordings can be an addition to debriefs because it provides caregivers with more detailed feedback of their performance. Nadler et al. (21) showed that debriefs supported by recordings improved teamwork at subsequent resuscitations. Nevertheless, when caregivers know they are recorded, the Hawthorne effect may occur: behavior can be changed because they are aware they are being observed. However, this is usually a change for the better (17, 24).

Multiple studies have been carried out which try to improve quality of care by using recordings for video-reflection with the caregivers. Scherer et al. (25) showed that guideline compliance improved more when video recording was used to reflect on Advanced Trauma Life Support (ATLS) when compared to verbal feedback. Other aspects in trauma care, besides guideline compliance, that improve after video-reflection are resuscitation technique, adherence to assigned tasks and a decreased variability between caregivers (3, 24, 26, 27). It is particularly important for pediatric trauma centers to maintain their ATLS skills as they treat critically injured patients with a lower frequency than adult trauma centers (24). The long term effect of video review has also been studied. Raphael et al. (28) showed video-reflection increased the compliance rate to the time-out process before an intervention. This improvement remained for at least 2 years.

In neonatal care, Root et al. studied a cohort of infants born before and after implementation of weekly video reflection and compared the recordings to the resuscitation guidelines. Providers complied more often to guidelines and correct documentation increased from 39 to 65% in the period after implementing weekly video-reflection sessions (29). Video-reflection is considered most effective in combination with skill training or quality improvement programs in improving technical as well as non-technical skills (12, 22). After a combination of a skill training program and video-debriefing was introduced, Skåre et al. (30) showed an improvement in time to effective spontaneous breathing and in compliance to guidelines. The ultimate goal of video-reflection is often improving patient outcomes by improving the practice of the caregiver. The perspective of the caregiver is, however, not often the objective of a study. It might be interesting to see the effect of video-reflection on for example their level of confidence or relaxation during interventions which ultimately also has an effect on patient care.

However, the catch with video-reflection is how to actually use the findings for education or improvement in a way that is effective and sustainable. It is recommended for future team trainings to implement the findings of video reflection in a feedback system and to follow the changes that occur over time (31). Data acquired from video review must be used to drive change, otherwise it becomes a mundane task (32). It is recommended to conduct video-review regularly, for example weekly, with a predefined timeslot and integrated in training and education to avoid a decline in skills and knowledge after it is learned (33). This will create a cycle of evaluation (by video-reflection), training and re-evaluation (30, 31, 33). However, future research is needed to assess different methods for sustainability. This can help to maximize the efficiency of video review (5).

## Using reflexivity to learn from everyday practice

While recordings can be used to focus an individual on their errors or non-compliance, there is also a different approach. Reflexivity uses recordings to enable caregivers to review recordings together to learn from interventions, context and conducts and collaboratively find ways to improve care (4, 34). Where reflection is personal and focused on specific aspects of caregivers own conduct, reflexivity is broader and focused on how implications of learning can impact the context in which they work together. Reflexivity is especially useful to improve quality of complex care. So far, methods such as simulation-based education and debriefing have been used to improve quality of care (35, 36). However, to learn from aspects of care that caregivers enact unconsciously, that are unexpected, or how every patient and intervention requires a different approach, real-life recordings are required (6). When showing these recordings to the front-line staff, caregivers can obtain different perspectives and their input can be used as the base for quality improvement. Reflexivity can be the next step improving quality of complex care (4, 37).

Hor et al. used reflexivity to design the space in an intensive care unit to maximize communication effectiveness. In the IC ward an open-plan design was used for accessibility. However, the open space gives more opportunities for interruptions as well. This study showed how video-reflexivity can be used to establish improvement as an ongoing process instead of a response to adverse events. They used solutions such as changes in behavior and reflection to improve communication (38). Reflexivity has also been used to explore the impact of health professionals on the facilitation of skin-to-skin contact within the first two hours after a caesarian section. A study by Stevens et al. sheds a light on the amount of contact between mothers and their babies, which was low, physically as well as emotionally. Here, video-reflection has been used to visualize care in a new way resulting in an increased understanding of what happens in practice. This kind of perspective is often overlooked or not paid attention to (39). Crenshaw et al. (40) described how video-feedback, reflection and interactive analysis could be an innovative approach to improve nurse leadership practices to reduce nursing shortage. Lastly, Sarcevic et al. examined leadership structures in a trauma center to identify weaknesses. As a diversity of patients with different injuries is admitted to trauma centers, effective leadership is essential to evaluate and treat each patient in the best way. In this study, video-reflexivity was used to identify leadership structures. Suggestions for technology design to support teamwork and patient care were made, e.g., using a smart-badge to enable more efficient role-identification or digital pen and paper technology (41).

In summary, video-reflexivity can be used to visualize care and social structures in new ways to drive continuous change and improvements. Especially in emergency care the environment can be chaotic. Through reflexivity caregivers can get a full overview of all the different aspects of emergent care and give input on improving the quality of care.

## Preconditions for the organization of video-reflection

Even though we live in a social media era where recording is part of everyday life, recording care is not yet a general practice (5, 42). Caregivers may find it difficult to record their practice and reflect on it with others. They may feel exposed, embarrassed and vulnerable because of the possibility of negative feedback on their behavior (43). Studies have shown that the solution is to engage the caregivers in the goal of video reflection and to obtain a feeling of trust and safety (44, 45). McNicholas et al. (45) hypothesized in their review that when caregivers have had a higher exposure to simulation or review training during their education, they had less anxiety when being recorded. However, those who feel uncomfortable when being recorded should be given the opportunity to have an alternative location or to control the activation of the cameras (44).

To activate the caregivers, Lloyd et al. recommends to generate short-term wins to create a sense of urgency. For example, when the implementation of a new checklist has improved verbal skills of the team leaders during intubation, this should be visible for the entire organization and celebrated (46). Dumas et al. (42) emphasized data of recordings should only be accessible to approved entities and be reviewed only at approved venues (e.g., with only peers present). Regardless, when a department has actually implemented video recordings, it is often accepted by caregivers who are participating (26). A study by den Boer et al. showed caregivers on the department of neonatology experience recording and reviewing neonatal resuscitation beneficial for learning and improving neonatal resuscitation skills (47). Shivananda et al. (48) showed a high rate of willingness and acceptance after implementation of video-review.

Still, common hurdles of video review that remain within a team are technical challenges like functioning of equipment and storing of videos. Other challenges are time and resource constraints, as it is time consuming to prepare video-reflection sessions and storage capacity needs to be considered carefully before the start of a video-reflection project (31, 49). There is still no existing or dedicated installation specifically designed for emergency medicine that enables recording and storing medical videos in a safe manner. Every hospital finds their own way in this.

Cultural differences may also play a role in implementing video-reflection, but so far no studies have analyzed this. However, studies have shown that ethical and legal issues play a role (26, 31, 45, 50). Different standards and formalities of ethical committees exist and differ between centers and countries, which results in a variation in implementation of video-reflection regarding consent, data acquisition and storage (33). As the focus of the recordings is not the patient, but the quality of care provided by the caregivers, the consent procedure nearly always includes consent from the caregivers. In emergency care, informed consent may be difficult to obtain from the patient. This can make it hard to get legal permission for recording. Studies that record without consent from the patient often make review of recordings part of a safety and quality assurance process where consent is deemed unnecessary (51). The question remains if this goal is more important than the patient’s right of privacy (5). When the sole goal of video reflection is quality assurance, the recordings may be partially protected from medicolegal consequences by protective acts. However, local regulations need to be considered carefully by each center when implementing video-reflection.

## Technical aspects and future developments

When comparing a camera to an observer who is present to assess care, the observer present can be biased or miss certain aspects because they need to take it all in at once. Observers watching a recording however, can assess communication and leadership independently from technical quality (16). However, assessment of recordings can still be difficult. For example, Oakley et al. found that it was challenging to accurately assess the clinical state of the patient on recordings without complementary vital signs (9). Recordings offer only a restricted view of the context and effectiveness of interventions in emergency care. For example, Gelbart et al. (52) states that assessment of degrees of stimulation, color and Apgar scores are vulnerable to subjectivity, as is the context of the team functioning. When reviewing a video, one can be blind to aspects that are obvious to the team that was present (5).

As a solution, recordings can be made more all-encompassing by adding vital parameters that are monitored to the recordings. In neonatal resuscitation, a Respiratory Function Monitor (RFM) has been used to provide information on the physiological parameters of infants (53). Van Vonderen et al. (54) showed how the combination of the physiological parameters and recordings can improve quality and provide more useful feedback during review. The vital parameters and the recording complement each other and visualize the effect resuscitation procedures achieve (55). This could be beneficial to interpretation of recordings during video-reflection.

Another method to make recordings more comprehensive is to add recordings of eye-tracking glasses. These give a unique point of view, e.g., the healthcare providers’ gaze. Wagner et al. showed that it is feasible, comfortable and has educational benefits. It can even be used for live time streaming to observers that are not present in the same room (56). Larsen et al. (57) showed it is effective for conducting research. In pediatric trauma simulation, research on eye-tracking showed it is feasible to use (58). Also, Dewar et al. investigated the use of body-worn cameras. Healthcare workers did not think it negatively interfered with carrying their job. It could also be a beneficial tool to analyze team performance (59).

Technology continues to develop and recordings are made more all-encompassing by adding vital parameters of the patient. This helps to understand complex care and team challenges. In trauma care, a so called Trauma Black Box has been developed. A black box from aviation contains a Flight Data Recorder where dozens of measurements from the airplane are stored combined with voice recordings from the cockpit. In emergency care, combined recordings of sound, video and vital parameters can also create a ‘flight recorder,’ e.g., it showcases a detailed recollection of what exactly went down during an intervention. This novel technology can be used to drive change and improve patient safety in the future (60). A different approach is using a video-based framework analysis to identify for example latent safety threats to patient safety during (61, 62). Fitzgerald et al. (63) even showed how recordings can help generate real-time algorithms to improve trauma resuscitation by analyzing preventable mortality and morbidity.

## Putting it into practice: a case-study of the neonatal intensive care unit (NICU) at Leiden University medical center (LUMC)

**FIGURE 1:**
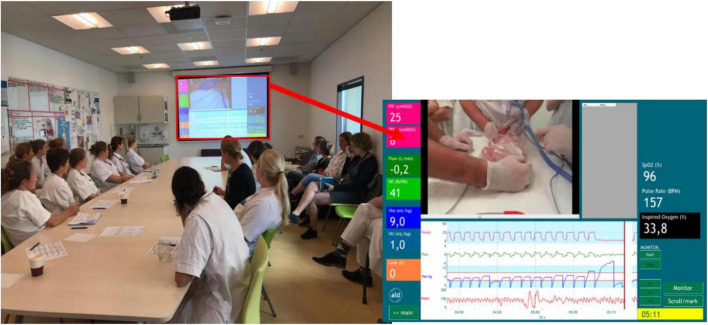
Video-reflection of a neonatal resuscitation on the Neonatal Intensive Care Unit (NICU) of the Leiden University Medical Center (LUMC) from 2014 until 2021. Recordings of the hands of the caregivers and the Respiratory Function Monitor (RFM) are visible.

Video and physiological parameter recording of neonatal stabilization was implemented at the Neonatal Intensive Care Unit (NICU) of the Leiden University Medical Center (LUMC) in 2009. Since 2014 weekly and plenary review sessions are held where recordings of neonatal stabilization are reviewed, focused on mostly on technical aspects (Figure 1). Recording and reviewing resuscitation is considered highly beneficial in our department for learning and improving resuscitation skills and is recommended by participating providers (64). It has proven to be a valuable tool for asking and giving objective feedback and to have educational benefits (29, 47). The recurring review sessions were said to enhance integration of lessons learned in the daily routine and improve patient safety (8, 47). Reviewing of neonatal interventions has become part of standard care in our department in the last 8 years and caregivers are used to recording their practice and to reflect on it on a regular basis. Recordings of neonatal stabilization are even reviewed with parents, who consider this to be valuable and reported positive experiences (65). Currently, caregivers even ask for recordings of their interventions to be reviewed.

**FIGURE 2:**
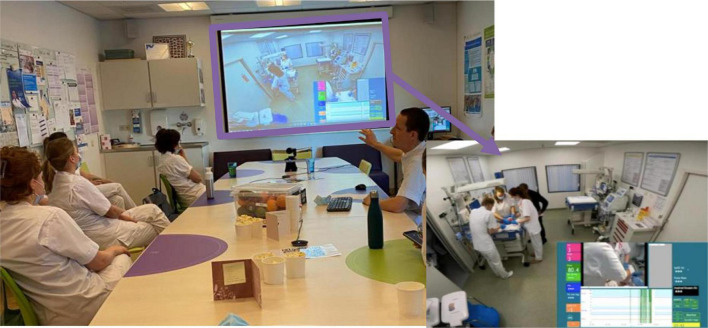
Video-reflection of a neonatal resuscitation from 2021 onward. Recordings of the hands of the caregivers, the respiratory function monitor and the resuscitation room are visible, with addition of audio. During the video-reflexive sessions, the focus is on the context.

**FIGURE 3:**
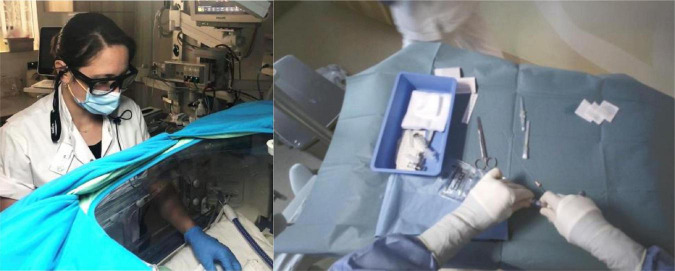
Use of the Tobii eye-tracking glasses on the Neonatal Intensive Care Unit (NICU)of the Leiden University Medical Center (LUMC), which give a unique point-of-view recording.

Our department want to continue using video recordings to improve patient care and safety in new ways. Using the insights from this review, we propose a set-up for a future study on the implementation of video-reflection and using it to improve quality of care (Figure 2). The focus of the reflection sessions will be on daily neonatal interventions and their context, on reflecting multidisciplinary instead of individual assessment and on interacting with the members from both medical and nursing staff to retrieve their perspective. We use eye-tracking glasses (Figure 3) to have an extra point of view and we have started recording intubations and sterile line insertions. Neonatal resuscitation will be recorded with multiple camera’s, including a camera showing the hands of the caregivers and the infant, next to a camera with room-view (Figure 2). To evaluate non-technical skills we will also add audio to our recordings. Combining these recordings will allow for reflection of technical aspects as well as non-technical aspects. We will implement findings that are derived from the weekly reflexive sessions. Also, we will investigate the short and long term benefits for patients and the quality of care and collect feedback from caregivers. Our goal is to discover how to make implementation of findings continuous and sustainable, as this has not yet been studied. Lastly, we will write a manual for other departments who wish to start using video-reflection. We aim to describe the success factors and boundaries that need to be overcome to maximize the potential video-reflection has to offer.

## Summary and future perspective

Video recording in emergency care has developed in many ways in the past years. It has been used as a tool for research and gives an objective view on work-as-done. Also, it has been used to improve care by showing recordings to the caregivers themselves which enables reflection and reflexivity on their actions. Still, it remains unclear how video-reflection can be best used to improve care or integrated in education to drive change. Although reviewing recordings in emergency care has been studied, this is mostly on the effect on guideline adherence or clinical skills. The improvement for long term and short term outcomes for the patient and the caregiver are still subject of research. Therefore, we suggest future studies should focus on how video-reflection is best implemented and used to improve quality of care.

## Author contributions

VHee and RW drafted the initial version of the manuscript. All authors participated in critical revision of the manuscript for important intellectual content, approved the final manuscript as submitted, and agree to be accountable for all aspects of the work.
