# Supplementary material for: Video recording emergency care and video-reflection to improve patient care; a narrative review and case-study of a neonatal intensive care unit
Source: Front Pediatr. 2022 Aug 4;10:931055. doi: 10.3389/fped.2022.931055 (PMC9385994; doi:10.3389/fped.2022.931055)
Supplement: Supplementary file 1 [file Table_1.DOCX]

**Search strategy in PubMed (2021 October 7^th^)**

| **#** | **Query** | **Results** |
| --- | --- | --- |
| **#9** | #3 OR #5 OR #7 OR #8 | **1,707** |
| **#8** | "videoreflecti*"[tiab] OR "video reflecti*"[tiab] OR "video evaluation"[tiab] OR "videoevaluation"[tiab] | **79** |
| **#7** | #1 AND #6 | **132** |
| **#6** | "team training"[tiab] OR "teamwork training"[tiab] | **1,521** |
| **#5** | #1 AND #4 | **1,186** |
| **#4** | "Quality Improvement"[Mesh] OR "Quality Assurance, Health Care"[Mesh:NoExp] OR "quality improvement"[tiab] OR "quality assurance"[tiab] | **130,581** |
| **#3** | #1 AND #2 | **396** |
| **#2** | "Clinical Audit"[Mesh] OR "audit"[tiab] OR "audits"[tiab] OR "auditing"[tiab] OR "auditor"[tiab] OR "auditors"[tiab] OR "auditable"[tiab] | **54,063** |
| **#1** | "Video Recording"[Mesh:NoExp] OR "Videodisc Recording"[Mesh] OR "Videotape Recording"[Mesh] OR "video*"[tiab] | **156,915** |

**Search strategy in Embase.com (2021 October 7th)**

| **#** | **Query** | **Results** |
| --- | --- | --- |
| **#10** | #9 NOT 'conference abstract'/it | **3270** |
| **#9** | #3 OR #5 OR #7 OR #8 | **4926** |
| **#8** | 'videoreflecti*':ti,ab,de,kw OR 'video reflecti*':ti,ab,de,kw OR 'video evaluation':ti,ab,de,kw OR 'videoevaluation':ti,ab,de,kw | **139** |
| **#7** | #1 AND #6 | **221** |
| **#6** | 'team training':ti,ab,de,kw OR 'teamwork training':ti,ab,de,kw | **2149** |
| **#5** | #1 AND #4 | **3827** |
| **#4** | 'total quality management'/exp OR 'health care quality'/de OR 'quality improvement':ti,ab,de,kw OR 'quality assurance':ti,ab,de,kw | **367218** |
| **#3** | #1 AND #2 | **977** |
| **#2** | 'clinical audit'/exp OR 'audit':ti,ab,de,kw OR 'audits':ti,ab,de,kw OR 'auditing':ti,ab,de,kw OR 'auditor':ti,ab,de,kw OR 'auditors':ti,ab,de,kw OR 'auditable':ti,ab,de,kw | **103689** |
| **#1** | 'videorecording'/exp OR 'videorecording' OR 'video*':ti,ab,de,kw | **230661** |

**Search strategy in Emcare (via Ovid ;2021 October 7th)**

| **#** | **Query** | **Results** |
| --- | --- | --- |
| **#9** | 3 or 5 or 7 or 8 | **1,566** |
| **#8** | ("videoreflecti*" or "video reflecti*" or "video evaluation" or "videoevaluation").mp. | **47** |
| **#7** | 1 and 6 | **100** |
| **#6** | ("team training" or "teamwork training").mp. | **1,201** |
| **#5** | 1 and 4 | **1,270** |
| **#4** | exp total quality management/ or health care quality/ or "quality improvement".mp. or "quality assurance".mp. | **135,745** |
| **#3** | 1 and 2 | **223** |
| **#2** | exp clinical audit/ or "audit".mp. or "audits".mp. or "auditing".mp. or "auditor".mp. or "auditors".mp. or "auditable".mp. | **28,021** |
| **#1** | videorecording/ or "video*".mp. | **69,576** |
